# The tissue origin of human mesenchymal stem cells dictates their therapeutic efficacy on glucose and lipid metabolic disorders in type II diabetic mice

**DOI:** 10.1186/s13287-021-02463-x

**Published:** 2021-07-07

**Authors:** Yinzhong Ma, Lisha Wang, Shilun Yang, Dongyu Liu, Yi Zeng, Lilong Lin, Linhui Qiu, Jiahao Lu, Junlei Chang, Zhihuan Li

**Affiliations:** 1 SIAT-GHMSCB Biomedical Laboratory for Major Diseases, Dongguan Enlife Stem Cell Biotechnology Institute, Dongguan Avenue 430, Dongguan, Guangdong China; 2 Shenzhen Key Laboratory of Biomimetic Materials and Cellular Immunomodulation, Institute of Biomedicine and Biotechnology, Shenzhen Institute of Advanced Technology, Chinese Academy of Sciences, Xueyuan Blvd 1068, Shenzhen, Guangdong China

**Keywords:** Mesenchymal stem cells, Diabetes, Hyperglycemia, Insulin resistance, Lipid metabolism

## Abstract

**Background:**

The therapeutic efficacy of mesenchymal stem cells (MSCs) of different tissue origins on metabolic disorders can be varied in many ways but remains poorly defined. Here we report a comprehensive comparison of human MSCs derived from umbilical cord Wharton’s jelly (UC-MSCs), dental pulp (PU-MSCs), and adipose tissue (AD-MSCs) on the treatment of glucose and lipid metabolic disorders in type II diabetic mice.

**Methods:**

Fourteen-to-fifteen-week-old male C57BL/6 *db/db* mice were intravenously administered with human UC-MSCs, PU-MSCs, and AD-MSCs at various doses or vehicle control once every 2 weeks for 6 weeks. Metformin (MET) was given orally to animals in a separate group once a day at weeks 4 to 6 as a positive control. Body weight, blood glucose, and insulin levels were measured every week. Glucose tolerance tests (GTT) and insulin tolerance tests (ITT) were performed every 2 weeks. All the animals were sacrificed at week 6 and the blood and liver tissues were collected for biochemical and histological examinations.

**Results:**

UC-MSCs showed the strongest efficacy in reducing fasting glucose levels, increasing fasting insulin levels, and improving GTT and ITT in a dose-dependent manner, whereas PU-MSCs showed an intermediate efficacy and AD-MSCs showed the least efficacy on these parameters. Moreover, UC-MSCs also reduced the serum low-density lipoprotein cholesterol (LDL-C) levels with the most prominent potency and AD-MSCs had only very weak effect on LDL-C. In contrast, AD-MSCs substantially reduced the lipid content and histological lesion of liver and accompanying biomarkers of liver injury such as serum aspartate transaminase (AST) and alanine aminotransferase (ALT) levels, whereas UC-MSCs and PU-MSCs displayed no or modest effects on these parameters, respectively.

**Conclusions:**

Taken together, our results demonstrated that MSCs of different tissue origins can confer substantially different therapeutic efficacy in ameliorating glucose and lipid metabolic disorders in type II diabetes. MSCs with different therapeutic characteristics could be selected according to the purpose of the treatment in the future clinical practice.

## Introduction

Type 2 diabetes mellitus (T2DM) is characterized by a series of metabolic disorders, including hyperglycemia, insulin resistance, dyslipidemia, and nonalcoholic fatty liver disease (NAFLD), which collectively contribute to the high mortality and morbidity of T2DM. T2DM accounts for 90–95% of diabetes mellitus cases, affecting almost 463 million individuals worldwide [1, 2]. At present, treatments for T2DM include diet control, exercise, oral hypoglycemic drugs, and insulin supplementation. However, these treatment methods can neither reconstruct the physiological blood sugar homeostasis of the body, nor avoid the risks of severe hypoglycemia and long-term adverse complications [3, 4].

Advances in stem cell biology have seen the rise of an exciting new field of research known as regenerative medicine. In the last few years, stem cell therapy has received wide attention as a treatment for chronic disease with complex symptoms, such as the central nervous system diseases, ischemic heart diseases, and autoimmune diseases [5]. Among various cell types that have been used, MSCs are considered to be one of the most promising types of stem cells for translational application because of their rich tissue sources, multilineage differentiation capacity, easy amplification in vitro, low immunogenicity, and unique immune biological properties [6, 7]. As a heterogeneous subset of stromal stem cells, MSCs can be isolated from various adult tissues, such as bone marrow, mobilized peripheral blood, umbilical cord Wharton’s jelly, dental pulp, and adipose tissue [8]. The diversity of tissue sources makes MSC superior to embryonic stem cells in terms of ethical concerns [9]. Based on these features, MSCs have become a very promising tool in regenerative medicine.

So far, several MSC-based drugs have been approved for clinical applications. For example, Cartistem (umbilical cord blood-derived MSCs) was approved for cartilage regeneration, and Prochymal (BM-MSCs) was approved for treating acute graft versus host disease [10, 11]. The versatile properties of MSCs have also attracted interests of clinicians as a potential therapy for diabetes. To date, 463 MSC studies for diabetes have been registered on clinicaltrials.gov [12]. These studies range in scope from diabetes-related vascular complications, to wound healing, and even include MSC therapy to treat new-onset diabetes. Promising results have been reported with the use of the MSCs regarding alleviation of hyperglycemia or dyslipidemia following T2DM. However, there is a lack of study to comprehensively compare the therapeutic properties of MSCs from different tissue sources for T2DM in the same experimental system. Some studies recommended the clinical application potential of adipose tissue-derived MSC (AD-MSC) or umbilical cord Wharton’s jelly-derived MSC (UC-MSC), while others concluded that dental pulp-derived MSC (PU-MSC) is the most optimal source. Here we performed a head-to-head study comparing the MSCs derived from human umbilical cord Wharton’s jelly, dental pulp, and adipose tissue in the *db/db* type II diabetic mouse model while uniformizing all study conditions regarding the mice’s gender, age, weight, housing conditions, and blood glucose level, in addition to, the cell count, phenotype, passage of cells, and the route of injection. This study will provide a full comparative assessment for the effectiveness of MSCs derived from these three sources in T2DM treatment.

## Materials and methods

### Animals and experimental design

Fourteen-to-fifteen-week-old male *db/db* mice and wild-type mice were purchased from Shanghai Model Organisms Center, Inc. All mice were housed individually with a 12-h light/dark cycle at 23 ± 2 °C and a humidity of 55 ± 10%, with free access to water and food. The mice were allowed to adapt to these conditions for 1 week prior to the experimental procedures. The animal procedures were approved by the Institutional Animal Care and Use Committee at Shenzhen Institute of Advanced Technology, Chinese Academy of Sciences. All related facilities and experimental procedures were performed according to the guidelines described by the National Institutes of Health guidelines for use and care of live animals.

The body weights of mice were firstly measured and then mice were assigned into 6 groups to make sure body weights were similar across groups: *db/db* + vehicle (n = 6), *db/db* + UC-MSCs (n = 18), *db/db* + PU-MSCs (n = 18), *db/db* + AD-MSCs (n = 18), *db/db* + Metformin (n = 6; 300 mg/kg, *p.o.*, cat. # M107827, Aladdin).

According to the recommendation of the International Society for Cellular Therapy, intravenous MSCs should be administered less than 2 × 10^6^ cells/kg body weight of humans, which is equal to 1.2 × 10^6^ cells/50 g body weight of MSCs for mice, according to the conversion equation based on body surface area (BSA) scale [13]. Therefore, based on the commonly used dosage by previous similar studies that investigated human MSCs in various mouse disease models [14, 15], the three MSC groups were further divided into three subgroups based on the numbers of injected cells (n = 6 per subgroup): 0.25 × 10^6^ (low dose, L) 0.50 × 10^6^ (medium dose, M), 1.00 × 10^6^ (high dose, H) cells per mice (50.2 ± 4.2 g). The MSCs (passage 5) were resuspended in 150 μl normal saline and administered by tail vein injection. The other groups were given an equivalent volume of vehicle saline as the wild-type + vehicle and *db/db* + vehicle groups, and all groups were fed a normal chow diet. To determine the effect of MSCs, fasting blood glucose, body weight, and the levels of serum insulin were measured before the cell injection treatments. Glucose tolerance tests (GTT) and insulin tolerance tests (ITT) were also performed before the cell injection treatments to determine the baseline tolerances of glucose and insulin. The protocols of GTT and ITT are detailed in the following sections.

### Isolation and culture of human MSCs

The MSCs used in this study were derived from human umbilical cord Wharton’s jelly (UC), dental pulp (PU), and adipose tissue (AD). The experimental protocols were approved by collaborative hospitals and obtained donors’ written consents. The human tissues used in this study were usually disposed as medical wastes and did not require any additional procedures performed on human subjects. In the current study, each kind of MSCs were derived from a single male donor that were chosen randomly from our cell repository. To ensure the quality of cells, donors were pre-screened for infectious diseases such as hepatitis B virus, hepatitis C virus, human immunodeficiency virus, cytomegalovirus, syphilis, and no family history of diseases (hematological diseases, metabolic diseases, chromosomal abnormalities, immunodeficiency diseases). UC-MSCs and PU-MSCs each were obtained from the donors with ages between 20 and 30. The PU-MSCs was prepared from the deciduous teeth from a donor randomly selected from 30 donors with ages between 8 and 15. Cells were amplified after being thawed and undertook a series of tests for quality control: the expression of cell surface markers, ability to proliferate and differentiate into osteogenic and adipogenic lineages.

Umbilical cord was collected from healthy donor mothers after their full-term delivery and immediately washed with normal saline for several times to remove red blood cells. After careful removal of these blood vessels, Wharton’s jelly tissues were chopped into small pieces of 0.5–1.5 mm^3^ and plated on the 75-cm^2^ cell culture flask for 12 h, followed by being covered with α-MEM medium (cat. # C12571500BT, Gibco) containing 10% Human platelet lysate (HPL, cat. # HPCFDCRL50, HELIOS). The medium was changed every 5 days. Cells were passaged at 90% confluence by digestion with TrypLE (cat. #12604021, Gibco). UC-MSCs were passaged till to passage 5 to be used in subsequent experiments.

Dental pulp tissues were obtained from shed primary teeth of children aged between 8 and 15 years with informed consents from their parents. The tissues were minced and digested in 10 times the volume of 0.3% collagenase type II (cat. # C6885-1G, Sigma-Aldrich) at 37 °C for 30 min. Subsequently, the α-MEM containing 5% HPL was added to terminate digestion, followed by centrifugation at 1200 rpm for 5 min and another addition of 5 times the volume of α-MEM containing 10% HPL which was then transferred to 25-cm^2^ cell culture flask and cultured at 37 °C, 5% CO_2_ incubation. The medium was replaced every 3 days. Cells were passaged at 90% confluence by digestion with 0.25% trypsin-EDTA and cultured in complete medium made of α-MEM medium supplemented with 5% HPL from passage one. PU-MSCs at passage 5 were used in all the experiments.

Adipose tissue was obtained in the form of waste aspirate from liposuction surgery. Tissues were cleaned in normal saline for 3 times and then digested with collagenase type II in HEPES by incubation in a shaker at 37 °C for 30 min. The digestion was terminated by the addition of 5% HPL. The mixture was centrifuged at 1200 rpm for 5 min. Cells were resuspended in complete medium made of α-MEM medium supplemented with 5% HPL, and they were transferred to 25-cm^2^ cell culture flask. Cells were cultured in 37 °C at 5% CO_2_ atmosphere. Floating cells were removed at 24 h, and cell culture medium was replaced with fresh complete medium every 2–3 days. Cells were passaged at 90% confluence by digestion with 0.25% trypsin-EDTA (cat. #25200072, Gibco). AD-MSCs at passage 5 were used in all the experiments.

### In vitro differentiation of MSCs

AD-, PU-, and UC-derived MSCs were differentiated into adipocytes, osteoblasts, and chondrocytes at three passages as follows. MSCs from all sources were seeded into 12-well plates at 10,000 cells per well and were maintained in standard culture medium until 60% confluence. Cells were cultured in adipogenic, osteogenic, or chondrogenic induction medium (All from Cyagen Biosciences Inc.). Cells were stained on days 14, 19, and 21, respectively, to assess adipogenic, chondrogenic, and osteogenic differentiation. Cells were fixed in 4% paraformaldehyde. Cells were stained with Oil Red O to assess adipogenic differentiation. To assess osteogenic differentiation, cells were stained with Alizarin red S. Cells were stained with Alcian blue to assess chondrogenic differentiation (all stains were procured from Cyagen Biosciences lnc.). The stained plates were observed under an inverted phase contrast microscope (Primovert, Carl Zeiss), and the images were captured using Zen Light Version 2.1.2580 software.

### Flow cytometry analysis

P3 cells were digested and washed twice with PBS, and then suspended in PBS at a concentration of 5 × 10^5^ cells/mL. Fluorescein phycoerythrin (PE) conjugated monoclonal antibodies that recognize CD105, CD90, CD73, CD34, CD45, and HLA-DR (BD Biosciences) were added to the cell suspension of UC-MSCs and PU-MSCs, while antibodies that recognize CD105, CD90, CD73, CD44, CD45, and CD31 were added to the cell suspension of AD-MSCs. The mix was incubated in the dark at 4 °C. PE conjugated mouse IgG isotype antibodies (BD Biosciences) were used as negative controls. After 30 min incubation, MSC immunotypes were determined by a FACSAria flow cytometry (FS300, Amnis) and analyzed using the FlowJo software (IDEAS 6.2.187.0). The percentage of expressed cell surface antigen was calculated for 10,000 gated-cell events.

### Growth curve of MSCs

Cells were seeded into 12-well plates at 5000 cells per well and cultured. Cells were trypsinized and the cell number was directly determined on an automatic cell counter (IC1000, Countstar) every 24 h until cells were at 100% confluence. The cell growth curve was drawn with time as the abscissa and cell number as the ordinate.

### Intraperitoneal glucose tolerance test (GTT) and intraperitoneal insulin tolerance test (ITT).

After a 5 h fasting, an intraperitoneal ITT was performed on the mice to evaluate the effect of MSCs by previously described methods. Briefly, blood was first drawn from the tail vein once as time point 0, then the mice were intraperitoneally injected with insulin solution (1.5 U/kg) and the blood was collected at 15, 30, 60, 90, and 120 min for blood glucose determination with a blood glucose meter. After the ITT, mice were returned to the cage to rest with normal chow and water supply. To acquire a stable physiological state, GTT was performed at 48 h after the ITT. Briefly, blood was first drawn from the tail vein once as time point 0, then the mice were intraperitoneally injected with glucose solution (2 g/kg). Blood was collected at 15, 30, 60, 90, and 120 min for blood glucose determination. After the first round of cell injection, GTT and ITT were performed on the indicated groups to assess glucose and insulin tolerance every 2 weeks for a total of 4 times.

### Blood and tissue collection

At the end of the experiment, mice were injected intraperitoneally with 1% pentobarbital sodium (50 mg/kg) for anesthesia. Blood was collected and centrifugated at 3000 rpm for 15 min to obtain serum for biochemical analyses. Then, the mice were perfused through the left ventricle with 10–15 ml PBS, followed by 10–15 ml of 4% paraformaldehyde. After the perfusion, the liver was collected. One-half of the tissue was fixed overnight in 4% paraformaldehyde and then embedded in paraffin to make cross sections of 3 μm thickness for hematoxylin-eosin (HE) staining and Oil Red O staining. The HE staining was performed to assess hepatic injury, while the Oil Red O staining to detect hepatic lipid deposition.

### Biochemical analyses

The levels of alanine transaminase (ALT) (cat. #140120006, Mindray), aspartate aminotransferase (AST) (cat. #140220005, Mindray), Alkaline Phosphatase (ALP) (cat. #140320004, Mindray), total cholesterol (TC) (cat. #141620006, Mindray), triglyceride (TG) (cat. #140120006, Mindray), high-density lipoprotein cholesterol (HDL-C) (cat. #142120005, Mindray), and low-density lipoprotein cholesterol (LDL-C) (cat. #142020004, Mindray) in the serum were measured using standard analytics.

### Histopathological examinations

HE and Oil Red O staining were carried out following standard procedures. The HE and Oil Red O staining sections were photographed by a light microscope (OLYMPUS BX53). Quantitative analysis of tissue bubble and Oil Red signal in liver were performed using the image pro plus software.

### ELISA

The concentrations of serum insulin were determined using mouse insulin ELISA kits (cat. #CSB-E0571m, Cusabio).

### Statistical analysis

Data are represented as the mean ± standard error (SE) and analyzed using a one-way or a two-way ANOVA. P values were adjusted for multiple comparisons using Bonferroni correction. Analyses were performed using GraphPad Prism version 8 (GraphPad Software, Inc.). *P* < 0.05 was considered to be a statistically significant difference.

## Results

### Characterization of human MSCs derived from umbilical cord Wharton’s jelly, dental pulp, and adipose tissue

**Fig. 1:**
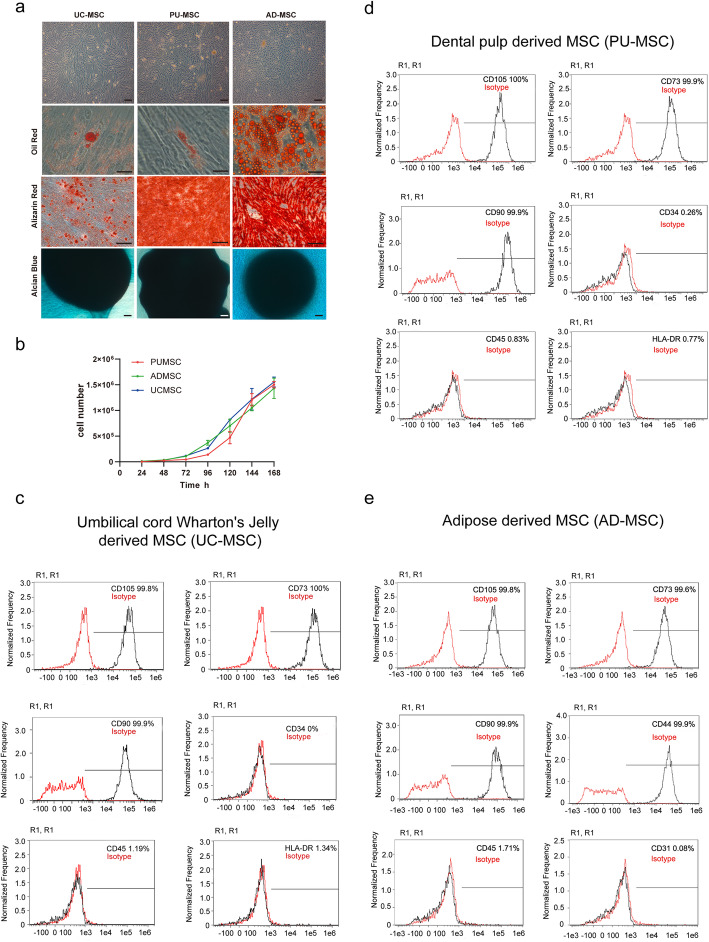
Morphology and multilineage differentiation of MSCs harvested from different tissue. **A** Representative micrographs of MSCs derived from umbilical cord Wharton’s jelly (UC), dental pulp (PU), and adipose (AD) observed under light microscopy. The MSCs appeared fibroblast-shaped. MSCs were able to differentiate into adipocytes identified by Oil Red O staining, osteocytes by Alizarin Red staining, and chondrocytes by Alcian blue staining. Scale bar = 50 μm. **B** Growth curves of MSCs and their cell viability. **C–E** Flow cytometric analysis of the expression of surface markers on MSCs. All three types of MSCs showed high expression of MSC-specific surface markers (CD105, CD73, CD90, or CD44), and low expression of hematopoietic cell marker (CD34), leucocyte marker (CD45), monocyte/macrophage marker (HLA-DR), or immune cell marker (CD31). Representative histograms of 3 independent experiments are shown

We first compared the phenotypes of MSCs isolated from different tissues by measuring their morphology, expression of cellular markers, and differentiation abilities. As shown in Fig. 1A, B, MSCs from human umbilical cord Wharton’s jelly (UC-MSC), dental pulp (PU-MSC), and adipose tissue (AD-MSC) were all fibroblast-like, attached to cell culture plates and shared a similar proliferating ability. To investigate the differentiation potential of the MSCs, the cells were directed toward the osteogenic and adipogenic lineages at P3. After 14 days of induction with adipogenic induction medium, cytoplasmic lipid droplets began to appear in AD-MSC abundantly, where a smaller amount of lipid droplets existed in UC-MSC or PU-MSC. The MSCs also possessed a chondrogenic and osteogenic phenotype by culturing with chondrogenic and osteogenic induction medium for 19 and 21 days, respectively, evidenced by Alcian blue and Alizarin Red staining. Therefore, it was suggested that all three MSC cell lines possessed the potential to differentiate into the three cell lineages mentioned above.

We next investigated the immunophenotype of three MSC lines at passage 3 by flow cytometry. A series of classical MSC phenotypic markers as defined by the International Society for Cellular Therapy criteria were examined, including CD44, CD34, CD105, CD45, CD31, CD73, CD90, and HLA-DR. As shown in Fig. 1C–E, all three MSC lines showed high expressions of MSC-specific surface markers (CD105, CD73, and CD90). Low expressions of hematopoietic cell marker (CD34), leukocyte marker (CD45), and monocyte/macrophage marker (HLA-DR) were showed in UC-MSC and PU-MSCs. Meanwhile, high expression of CD44 and low expression of CD31 (lymphatic and endothelial marker) were showed in AD-MSCs. The results above suggested that all three MSC lines retained their major phenotypes and differentiation abilities.

### Effects of MSC injection on blood glucose levels and glucose tolerance of *db/db* mice

**Fig. 2:**
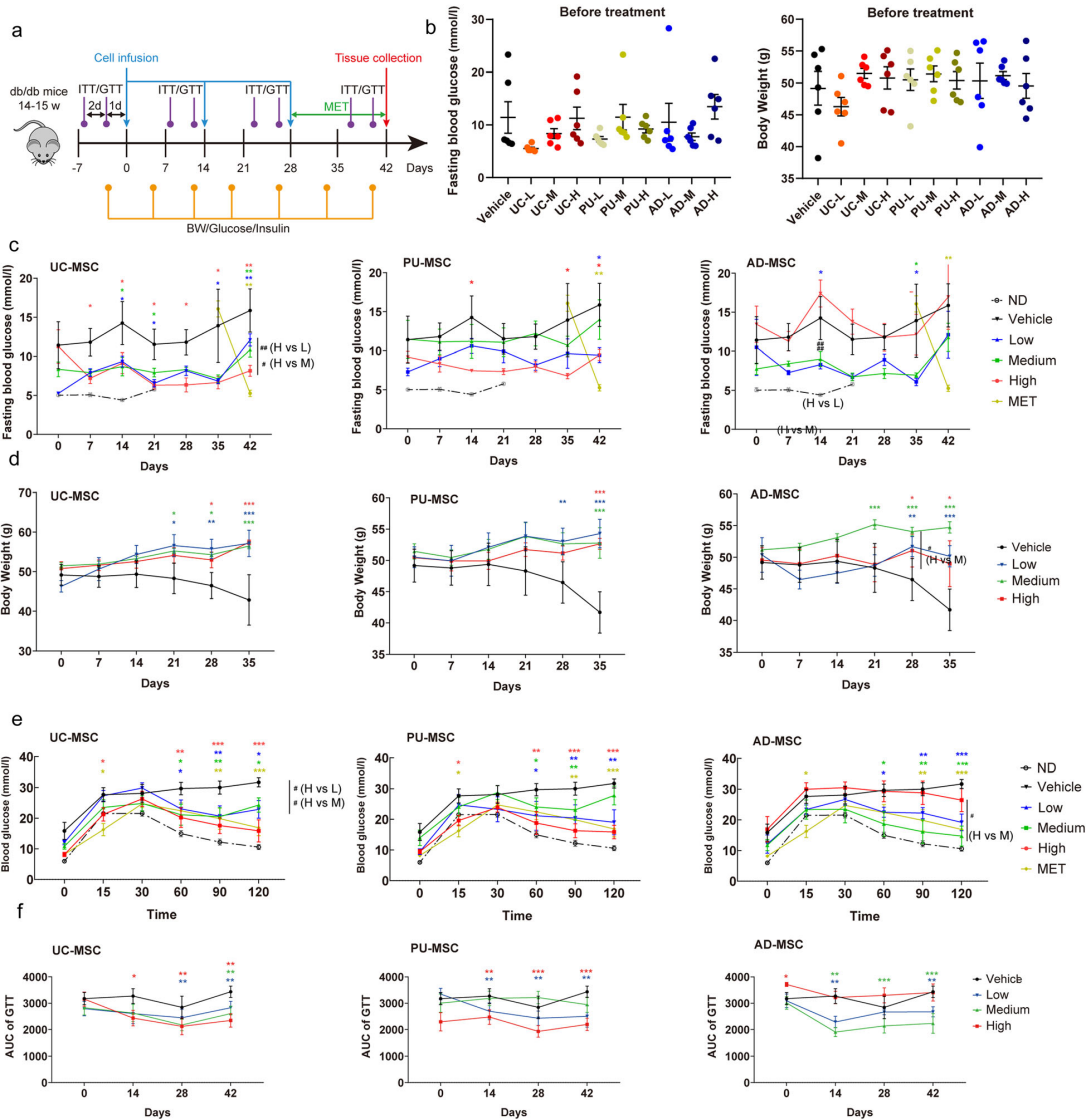
The effects of MSC injection on mouse body weights and blood glucose levels. **A** Experimental protocol for the MSC injection on *db/db* mice. *Db/db* mice of 14–16 weeks old were injected with human UC-MSCs, PU-MSCs, AD-MSCs, or PBS every 2 weeks (3 injections in 6 weeks). “L (low)”, “M (medium)”, and “H (high)” represent the doses of injected cells (0.25 × 10^6^, 0.5 × 10^6^, and 1.0 × 10^6^ cells per mice, respectively). Fasting blood glucose levels and body weights of the indicated groups were monitored every week during the whole experiment period. GTT and ITT was performed on the indicated groups to assess glucose tolerance and insulin sensitivity every 2 weeks during the whole experiment period. GTT was performed 2 days after ITT to achieve a stable physiological state of the animal. Metformin was given orally to animals in separate groups (MET) once a day at weeks 4 to 6. All the animals were sacrificed at day 42, and the tissue was collected for other indicated tests. **B** At the beginning of the experimental procedure, *db/db* mice were randomly assigned in indicated groups and the blood glucose levels and body weight were recorded. **C, D** Fasting glucose level and body weights were monitored during 6 weeks. The glucose level of the MET group in the three charts from **C** is derived from the same set of data. Non-diabetic (ND) wild-type mice were included as healthy controls. **E** Blood glucose levels from the last time of GTT among the indicated groups after three injections of MSCs. After fasting for 16 h, blood was collected from the tale vein (time point 0), then the mice were intraperitoneally injected with glucose solution (2 g/kg). Blood was collected at 15, 30, 60, 90, and 120 min for blood glucose determination. Non-diabetic (ND) wild-type mice were included as healthy controls. **F** AUC of all 4 times of GTT are illustrated among the indicated groups. All the results are expressed as the means ± S.E.M. n = 6 mice per group. ^*^P<0.05, ^**^P<0.01, ^***^P<0.001 vs vehicle. The colors of asterisks are consistent with the respective treatment groups and indicate the statistical significance versus the vehicle group. Significances between different doses of MSCs of the same tissue origin were indicated as ^#^*P*<0.05, ^##^*P*<0.01. Two-way ANOVA

Due to the dysfunction of the leptin receptor, *db/db* mice spontaneously develop hyperphagia-induced hyperglycemia, insulin resistance, and dyslipidemia. At baseline, the fasting blood glucose levels showed no significant difference among the groups (9.2 ± 2.4 mmol/l; Fig. 2A, B). Based on the doses of MSCs commonly used in mouse studies of metabolic diseases, three doses of cells (0.25 × 10^6^, 0.5 × 10^6^ and 1.0 × 10^6^ cells per mice) were used in the present study. After 3-time injections of MSCs, the *db/db* mice showed a dramatic decrease in the fasting blood glucose levels, while the PBS-treated *db/db* mice remained persistent hyperglycemic (12.92 ± 1.7 mmol/l; Fig. 2C). It is worth noting that there were differences in the efficacy of MSCs from the three tissue sources to regulate fasting blood glucose in *db/db* mice. A single infection of UC-MSC (1.0 × 10^6^ cells) significantly reduced fasting blood glucose in *db/db* mice and two injections of PU-MSC (0.25 × 10^6^ and 1.0 × 10^6^ cells) treatments achieved similar effects (Fig. 2C). Interestingly, only a trend of reduction of fasting blood glucose was observed after all the injections of medium dose (0.5 × 10^6^ cells) of PU-MSCs. Furthermore, no beneficial effect on fasting blood glucose was observed at the high dose of AD-MSC (1.0 × 10^6^ cells), but the low and medium doses (0.25 × 10^6^ or 0.5 × 10^6^ cells) could robustly reduce the fasting blood glucose after three injections (Fig. 2C). Therefore, the effects of PU- and AD-MSCs on fasting blood glucose was not strictly dose-dependent, which may be potentially due to variations within different groups of mice or the complexity of actions of that special tissue type of MSC in vivo.

Unlike what has been observed in some studies, the body weight of mice in the MSC-treated groups remained relatively stable, while the mice in vehicle group began to show significant weight loss since day 35 of the trial (Fig. 2D), which is similar to the clinical symptoms of severe diabetes due to impaired blood glucose absorption and storage.

Since a primary feature of *db/db* mice is glucose intolerance, we carried out glucose tolerance tests (GTT) after each time of MSC treatments. Starting from 30 min after the glucose injection, the blood glucose levels in the *db/db*-MSC group decreased significantly and the effects were comparable to metformin treatment (Fig. 2E), indicating a notable improvement in the glucose metabolism during the GTT test. We calculated the AUCs of all the GTTs and found that the ability of mice to maintain glucose homeostasis had been significantly improved by all three types of MSCs (Fig. 2F). Even a single dose of MSCs significantly improved the glucose tolerance of mice and this effect was maintained during continuous cell treatments. It is notable that different sources of MSCs require different doses of cells to confer therapeutic effects. The effective dose ranges for UC-MSC and PU-MSC were 0.5 × 10^6^ to 1.0 × 10^6^, while the AD-MSC was 0.25 × 10^6^ to 0.5 × 10^6^ cells per injection.

### Effects of MSC injection on insulin sensitivity and blood insulin levels in *db/db* mice

**Fig. 3:**
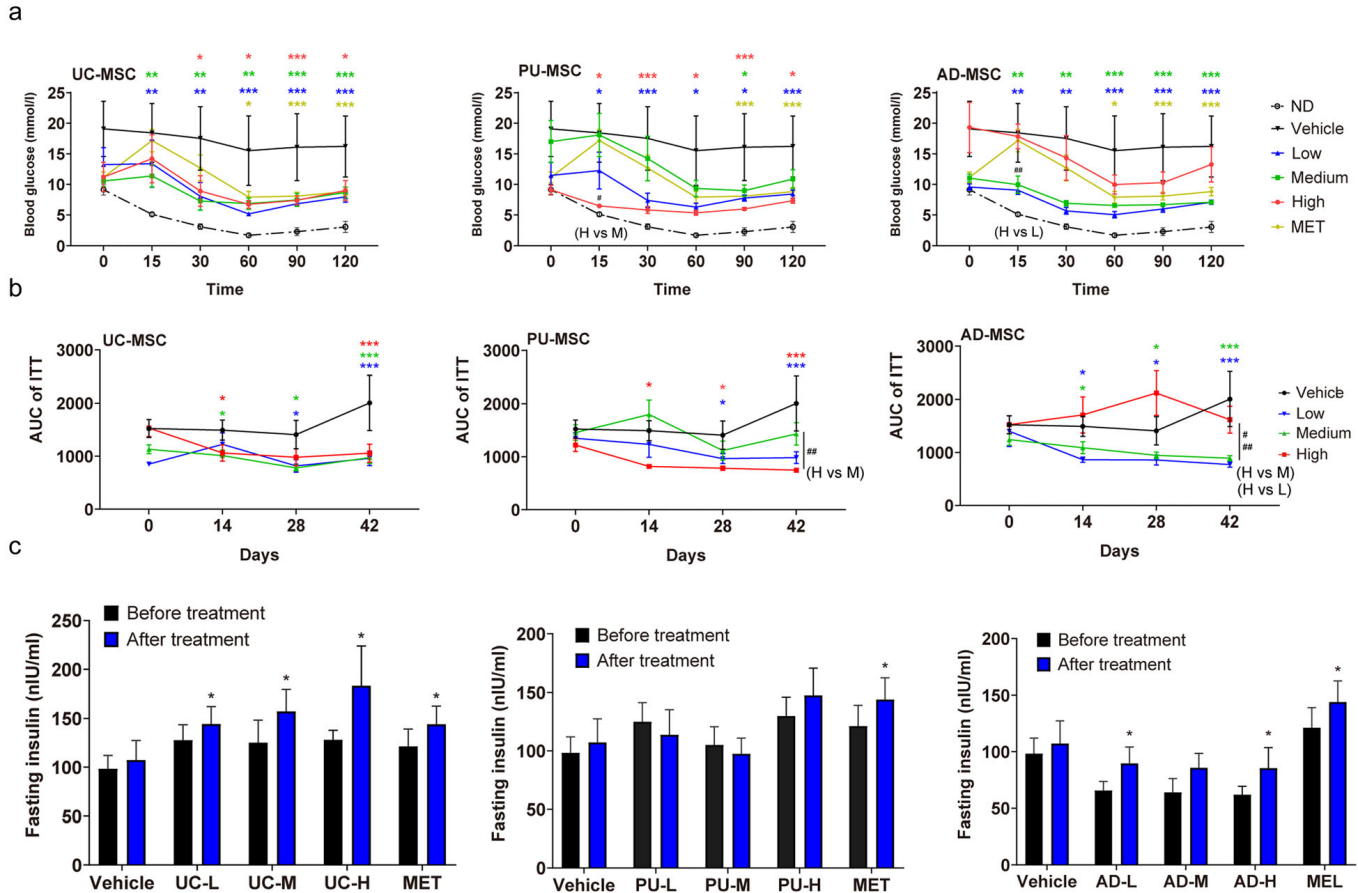
The effects of MSC injection on insulin sensitivity and blood insulin levels. **A** Blood glucose levels from the last time of ITT after three injections of MSCs. After fasting for 5 h, blood was collected from the tail vein (time point 0), then the mice were intraperitoneally injected with insulin solution (1.5 U/kg). Blood was collected at 15, 30, 60, 90, and 120 min for blood glucose determination. Non-diabetic (ND) wild-type mice were included as healthy controls. “L (low)”, “M (medium)”, and “H (high)” represent the doses of injected cells. MET, metformin. **B** ITT was performed on the indicated groups to assess insulin sensitivity, and the results were presented as AUC of all 4 times of ITT among the groups. **C** Fasting plasma insulin levels before and after the MSC injection (day 42) were determined by Elisa test. The results are expressed as the means ± S.E.M. n = 6 mice per group. ^*^P<0.05, ^**^P<0.01, ^***^P<0.001 vs vehicle. The colors of asterisks are consistent with the respective treatment groups and indicate the statistical significance versus the vehicle group. Significances between different doses of MSCs of the same tissue origin are indicated as ^#^*P*<0.05, ^##^*P*<0.01.Two-way ANOVA

In addition to glucose metabolism imbalance, *db/db* mice also showed insulin resistance as determined by ITT in this study. The blood glucose levels barely decreased after the injection of insulin in *db/db-*PBS mice. After 3 times of MSC injection (42 days since the first injection), mice receiving MSCs showed significantly faster blood glucose clearance during the ITT with effects even stronger than metformin treatment (Fig. 3A). Similar to the improvement of glucose tolerance, we also found that *db/db* mice showed better sensitivity to insulin after the first dose of MSC treatment, as shown by the results of AUC calculation (Fig. 3B). The effective dose ranges were 0.25 × 10^6^ to 1.0 × 10^6^, 1.0 × 10^6^, and 0.25 × 10^6^ to 0.5 × 10^6^ for UC-MSCs, PU-MSCs, and AD-MSCs, respectively (Fig. 3B). In addition to increase of insulin sensitivity, we also found that after 3 doses of UC-MSC or AD-MSC treatment, *db/db* mice showed higher levels of fasting plasma insulin compared to *db/db*-PBS controls (Fig. 3C).

Taken together, the results above suggested that all three types of MSCs improve the actions of insulin, whereas UC-MSCs and AD-MSCs also increase the plasma insulin levels.

### Effects of MSC injection on liver fat content and serum lipid profiles in *db/db* mice

**Fig. 4:**
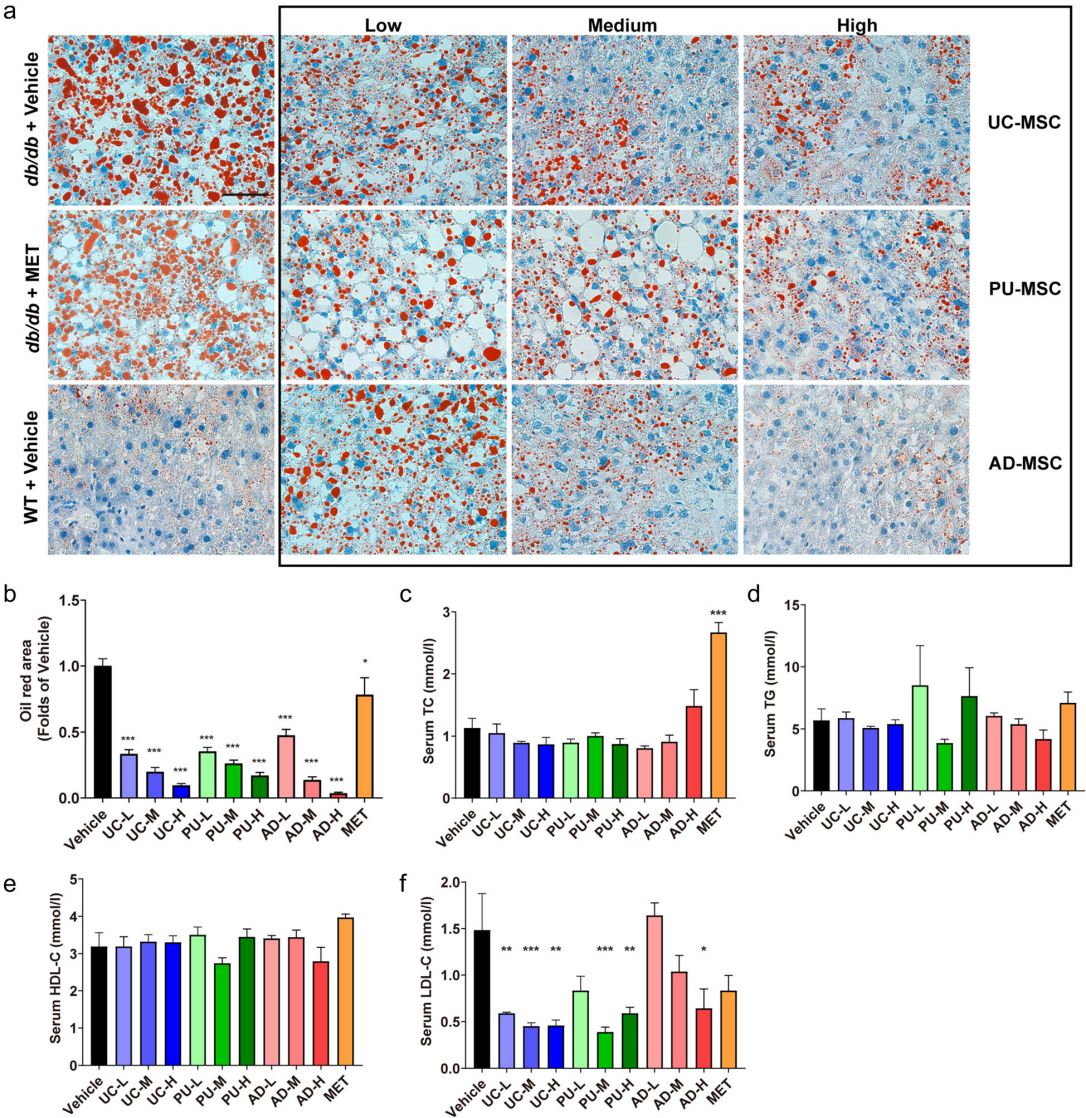
The effects of MSC injection on liver fat content and serum lipid profiles**. A, B** Representative images of Oil Red O-stained liver sections of the indicated groups. Scale bar = 50 μm. The red lipid staining were used for statistics. *db/db* mice were injected with human UC-MSCs, PU-MSCs, AD-MSCs, or PBS every 2 weeks for 6 weeks. MET, metformin. After the last injections, serum concentrations of **C** TC, **D** TG, **E** HDL-C, and **F** LDL-C in the indicated groups were measured. The results are expressed as the means ± S.E.M. n = 6 mice per group. ^*^P<0.05, ^**^P<0.01, ^***^P<0.001 versus the vehicle group. One-way ANOVA

Besides hyperglycemia and insulin resistance, nonalcoholic fatty liver disease (NAFLD) and dyslipidemia are also characteristic in *db/db* mice. To compare the hepatic fat changes, we performed Oil Red O staining on liver tissues. As shown in Fig. 4A, B, Oil Red O-positive area was obvious and widely distributed in the liver tissue of *db/db*-PBS mice, but showed a significant dose-dependent reduction in all three MSC treatment groups, indicating a dramatic decrease of lipid deposition in the liver after MSC treatment. Besides that, we measured the levels of serum lipids that characterize dyslipidemia to further explore the therapeutic effect of MSC injection. Our results showed that serum TG, TC, and HDL-C levels showed no significant change between *db/db*-MSC and *db/db*-PBS groups (Fig. 4C–E). In contrast, LDL-C levels in UC-MSC- and PU-MSC-treated groups were robustly decreased compared with *db/db*-PBS group with a relatively modest effect seen in the AD-MSC group (Fig. 4F). These data suggest that liver fat deposition and dyslipidemia could be alleviated by MSC treatments and the tissue origins of MSCs affected the efficacy.

### Effects of MSC injection on hepatic functional injury in *db/db* mice

**Fig. 5:**
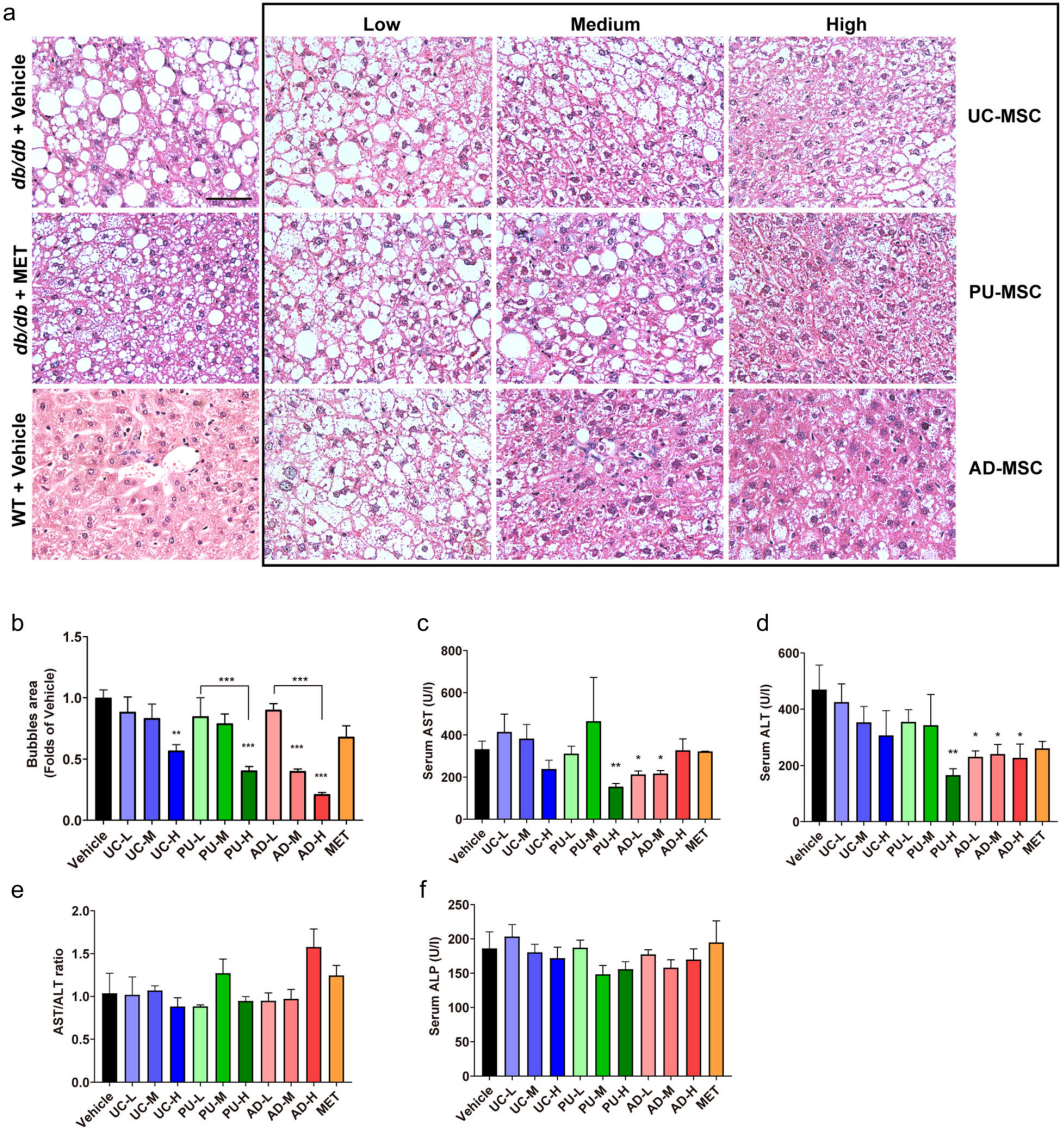
The effects of MSC injection on hepatic functional injury. **A, B** Representative images of HE-stained liver sections of the indicated groups. Scale bar = 50 μm. Hepatocellular ballooning in the liver of the indicated groups were scored. MET, metformin. After the last time of cell injection, serum concentrations of **C** AST, **D** ALT, **E** AST/ALT ratio, and **F** ALP were measured. The results are expressed as the means ± S.E.M. n = 6 mice per group. ^*^P<0.05, ^**^P<0.01, ^***^P<0.001 versus the vehicle group. One-way ANOVA

We next determined the impact of MSC injection on liver functional injury. HE staining showed normal hepatic lobules with regularly arranged hepatocytes and small lipid droplets in the control wild-type (WT) mice (Fig. 5A). The *db/db*-PBS group presented obvious steatosis, indicated by enlarged hepatocytes containing lipid droplets of various sizes, with a distinct hepatocellular ballooning and parenchymal steatosis. In contrast, treatment with UC-MSC (1.0 × 10^6^ cells), PU-MSC (1.0 × 10^6^ cells), or AD-MSC (0.5–1.0 × 10^6^ cells) displayed smaller-sized hepatocytes with remarkably alleviated ballooning injury (Fig. 5A, B). Additionally, functional hepatic injury was evaluated by determining the serum ALT and AST levels. The results displayed that the ALT and AST levels were 469.2 ± 87.6 and 332.7 ± 37.8 U/l in the *db/db*-PBS group (Fig. 5C, D), indicating impaired liver function of *db/db*-PBS mice. Consistent with previous reports, injections of AD-MSCs significantly decreased the elevated levels of ALT and AST in *db/db* mice (227.5 ± 48.5 U/l and 212.4 ± 16.2 U/l, respectively), suggesting that the hepatic functional injury was alleviated (Fig. 5C, D). Besides AD-MSCs, injection of high-dose PU-MSC (1.0 × 10^6^ cells) also induced a robust decrease in the levels of serum ALT and AST (Fig. 5C, D). AST to ALT (AST/ALT) ratio is a useful predictor of long-term complications such as liver fibrosis and cirrhosis [16, 17]. Similar to other research, we found that mice from *db/db*-PBS group presented an AST/ALT ratio that were approximate to 1.0 (1.03 ± 0.23), suggesting a moderate chronic hepatitis. Several MSC-treated groups showed decline of this ratio to less than 1.0 (UC-H, PU-H, PU-L, AD-M, and AD-L), suggesting the severity of chronic hepatitis was prone to being reduced although not significantly. Serum levels of another liver function biomarker ALP were not significantly altered by any type of MSCs used. Taken together, these data suggested that AD-MSC treatment is more effective in reducing functional injury in liver than UC-MSC and PU-MSC treatments.

## Discussion

**Fig. 6:**
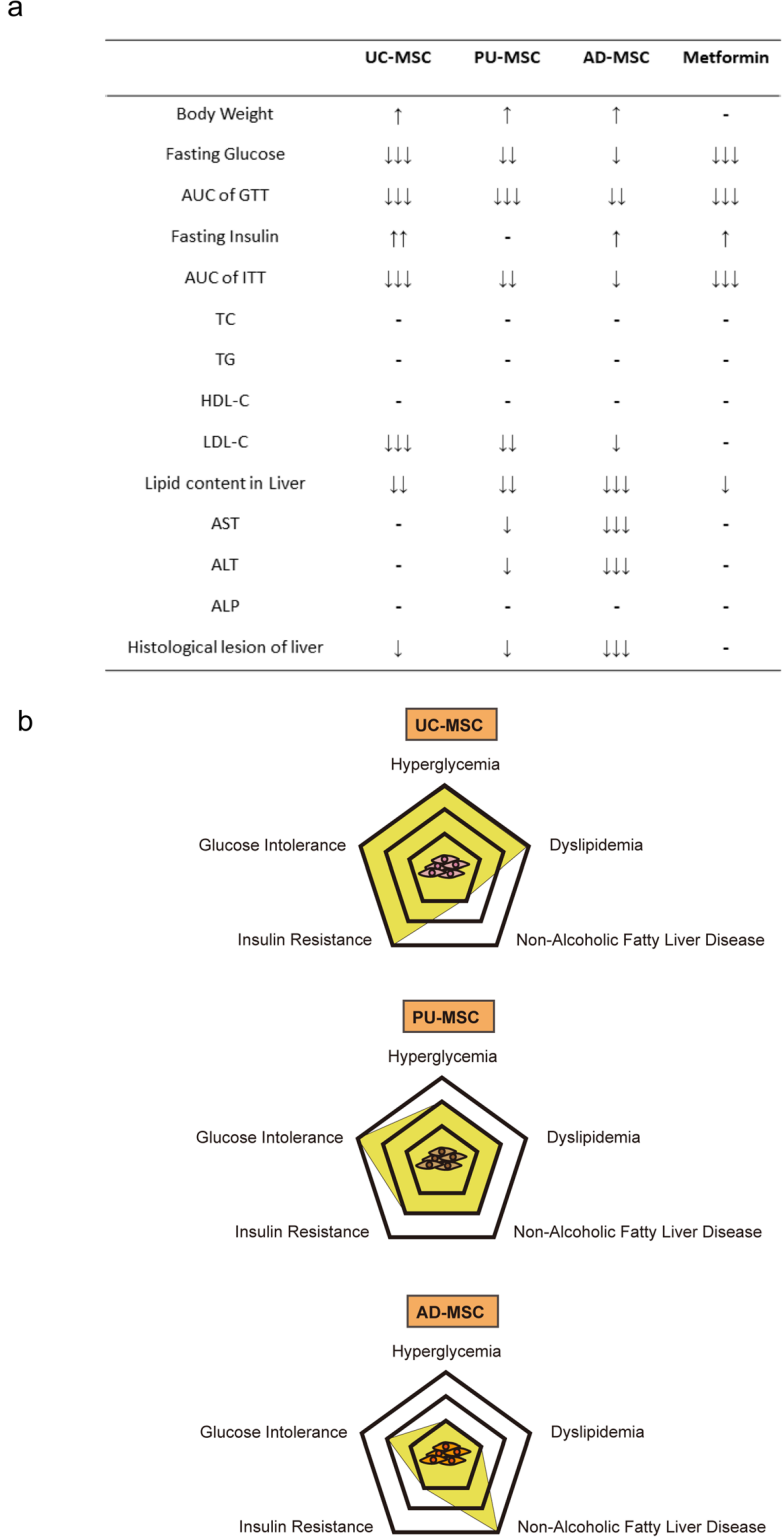
Summary of the beneficial effects of three types of MSCs in *db/db* mice. **A** Based on the experimental observations and statistical analysis, the therapeutic effects of three MSC cell lines on the presented diabetes pathologic indicators are summarized in the table. “↑↑↑” or “↓↓↓” means that the indicated treatment has a significant elevation or reduction on this index (^***^P<0.001). “↑↑” or “↓↓” means the observed therapeutical effect was inferior to “↑↑↑” or “↓↓↓”, but still significant on the index. “↑” or “↓” indicates that the indexes have only slight change compared with other groups. “-” means that no significant change were observed ( ^*^P>0.05). **B** Schematic diagram of MSCs derived from UC, PU, and AD on the improvement of diabetes pathology. “√” means that the indicated treatment had a significant therapeutic effect on the pathological symptoms. “-” stated that the relative treatment was not strong enough to significantly ameliorate the indicated symptoms

In this study, we comprehensively compared the phenotypes and therapeutic effects of MSCs harvested from human umbilical cord Wharton’s jelly, dental pulp, and adipose tissue in the treatment of glucose and lipid metabolic disorders in the *db/db* type II diabetic mouse model. Under the same experimental criteria, we found that UC-MSCs had the strongest ability to alleviate hyperglycemia, glucose intolerance, insulin resistance, and dyslipidemia, whereas AD-MSCs showed quite modest or no effects on these symptoms (summarized in Fig. 6). PU-MSCs displayed an intermediate efficacy among these three types of MSCs. In contrast, AD-MSC treatment significantly ameliorated hepatic functional injury, as evidenced by markedly decreased serum levels of ALT and AST and dramatic reduced histological lesion of liver tissue, which was only modestly improved by UC-MSC or PU-MSC treatment (Fig. 6). Our results demonstrated that MSCs of different tissue origins can confer substantially different therapeutic efficacy in treating various metabolic symptoms of type II diabetes.

The use of MSC in the treatment of metabolic diseases such as diabetes mellitus has been extensively undertaken in preclinical and clinical studies [18, 19]. The therapeutic efficacy of MSCs is highly linked to their physiological state, dose, and tissues where they are derived from. However, this has been largely overlooked in translating preclinical studies to the human clinical setting. Currently, umbilical cord Wharton’s jelly and adipose tissue-derived MSCs are the most commonly used in metabolic disorder research. UC-MSCs possess a unique combination of prenatal and postnatal stem cell properties and have higher proliferative potential and lower immunogenicity with allogeneic sources than other commonly used MSCs such as bone marrow-derived MSCs (BM-MSCs). Moreover, the noninvasive procurement and painless collection procedures make UC-MSCs a versatile candidate for clinical applications [20, 21]. It is worth mentioning that MSCs from umbilical cord blood and umbilical cord Wharton’s jelly demonstrated significance differences in efficacy on the treatment of T2DM [22]. Administration of MSCs from umbilical cord Wharton’s jelly resulted in significant improvement for glycemic homeostasis as well as β cell function in T2DM, while MSCs from umbilical cord blood showed a lack of significant therapeutic effects. In addition, UC-MSCs have been shown to alleviate insulin resistance and improve glucose homeostasis in high-fat diet/streptozotocin- (STZ-) induced T2DM rodent models [14, 16]. Meanwhile, UC-MSCs have been demonstrated to exert therapeutic effects on liver diseases, such as decompensated liver cirrhosis [17, 23], liver failure [24], and chemical-induced liver injury [25, 26]. In human clinical trials, injection of allogeneic MSCs harvested from the umbilical cord Wharton’s jelly or bone marrow into T2DM patients improved the function of pancreatic β cells, reduced the incidence of diabetic complications, and led to insulin independence in some patients [27–29].

Compared to UC-MSCs, AD-MSCs have become popular for therapy because of the ease of acquisition, high proliferative capacity, low immunogenicity, and high levels of multipotency. In cellular models, AD-MSCs have been shown to exert insulin-sensitizing effects via the restoration of insulin-stimulated glucose uptake [30]. AD-MSCs can also ameliorate lipid metabolic disturbance and improved glucose homeostasis in diet-induced obesity (DIO) mouse models [31, 32]. In rodent T2DM models, injection of human AD-MSCs improved glucose tolerance, preserved pancreatic β mass, and increased β cell proliferation [31, 33, 34].

More recently, the human dental pulp-derived mesenchymal stem cells (PU-MSCs) have emerged as a promising source of stem cells in regenerative treatments because they are less expensive to acquire and easier to harvest than other MSCs which require the costly and invasive techniques to isolate. PU-MSCs usually come from deciduous teeth, third molars, extra or orthodontic teeth, without adversely affecting the health of permanent dental pulp tissue, and there is no ethical controversy in research and development applications. Similar to the other MSCs mentioned above, PU-MSCs also exhibit self-renewal, multilineage differentiation potential, and immunomodulatory properties. Although PU-MSCs have not yet been tested directly in animal models of diabetes mellitus, the potential advantages of PU-MSC-based approaches for T2DM treatments have been proved. Recent studies have demonstrated that PU-MSCs were capable of differentiating into pancreatic cell lineage resembling islet-like cell aggregates (ICAs) with the ability to produce insulin [35–37]. Subcutaneous transplanted ICAs differentiated from PU-MSCs restored normoglycemia [38].

Although emerging data from animal studies have demonstrated that injection of MSCs reduces blood glucose levels and restores lipid metabolic balance, the features of MSCs derived from different sources vary greatly. In the present study, when comparing the MSCs derived from different sources, we found that a single dose of UC-MSCs significantly reduced fasting blood glucose levels in *db/db* mice. After the 6 weeks treatment (3 times of cell injection), both UC-MSCs and PU-MSCs treated mice demonstrated a significant blood glucose-reducing effect. This was associated with improved glucose tolerance, indicating that both UC-MSCs and PU-MSCs possess strong beneficial effects on glucose homeostasis. Although AD-MSCs were inferior to UC-MSCs and PU-MSCs in terms of controlling hyperglycemia, it still possesses blood glucose-reducing effect and therefore might be used for the treatment of metabolic syndrome, since AD-MSCs also showed other beneficial effects in *db/db* mice.

Insulin resistance is a key feature of T2DM. *Db/db* mice have been characterized by insulin resistance that mimics the natural progression of T2DM in human [28, 32]. In line with other research, we found that injection of UC-MSCs or AD-MSCs improved plasma insulin levels. Interestingly, although PU-MSC had no direct effect on plasma insulin levels, it improved insulin sensitivity efficiently in *db/db* mice, suggesting a potential therapeutic role that deserves further study.

Dyslipidemia and nonalcoholic fatty liver disease (NAFLD) are common complications of obesity-induced T2DM and play a critical role in the progression of liver functional injury. According to previous studies, injection with either UC-MSCs or AD-MSCs can ameliorate liver injury and restored lipid metabolism [14, 28, 32]. In agreement with other studies, our data further confirmed that UC-MSCs and AD-MSCs could significantly reduce lipid deposition in the liver in a dose-dependent manner. Besides that, we found that PU-MSCs can also reduce lipid deposition in the liver, despite their efficiency is slightly inferior to that of AD-MSCs and UC-MSCs. LDL-C is a type of lipoprotein that carries cholesterol into peripheral tissue cells. The content of LDL-C is correlated with the incidence and degree of cardiovascular disease. Among the serum lipids measured in this study, LDL-C was the only one that showed significant reduction after MSC treatment; to be specific, the UC-MSC and PU-MSC treatment, which further demonstrates the efficacy of MSCs derived from different tissue, can be varied drastically.

The therapeutic effects of stem cell transplantation on liver fibrosis and cirrhosis have been widely investigated in mice and humans. Serum transaminase (ALT and AST) activities are well known markers of such injury. In contrast to other studies showing that injection of UC-MSCs markedly decreased serum levels of ALT and AST, our data suggest that only AD-MSCs have a significant effect, as evidenced by alleviated histological lesion of liver tissue in *db/db* mice. Although there has no direct evidence that PU-MSCs have an impact on NAFLD, it has been proved that PU-MSCs had the capacity to differentiate into functional hepatic cells [35, 37]. Our data showed for the first time that high doses of PU-MSCs also significantly reduced plasma ALT and AST levels to the extent similar to those of AD-MSCs.

Since MSCs used in this study were obtained from different donors, we cannot rule out the possibility that differences of the therapeutic efficacy of MSCs from different tissues could be related to a specific characteristic of the donor, such as genetic background, life style, and undetermined physical parameters. We have predetermined strict criteria for donor selection in this study to minimize the differences in cell status between MSCs from different donors as much as possible, given that it is practically impossible to obtain umbilical cord, adipose tissue, and dental pulp from one person. Further study may be needed to clarify the influence of various physiological characteristics of donors on the therapeutic efficacy of MSCs with the same tissue origin.

## Conclusions

Taken together, these results demonstrate that all three types of MSCs are considered effective sources for treating T2DM and related metabolic syndromes but with quite differential effects on various symptoms. UC-MSCs exert stronger effects on improving hyperglycemia, glucose intolerance, insulin resistance, and dyslipidemia, where AD-MSCs are more effective to reduce liver fat content and functional injury. Furthermore, we demonstrate for the first time that PU-MSCs can also reduce hyperglycemia, serum LDL-C levels, and liver lipid deposition and restore insulin sensitivity to an extent similar to that of UC-MSCs. High-dose PU-MSCs can play a similar role to AD-MSCs in ameliorating liver functional injury. Detailed comparisons are shown in Fig. 6. MSCs derived from various tissue sources possess different therapeutic characteristics and therefore must be selected according to the symptoms of disease and purpose of the treatment in the future clinical practice.

## Data Availability

The data that support the findings of this study are available from the corresponding author upon reasonable request.

## Abbreviations

MSCs: Mesenchymal stem cells
MET: Metformin
GTT: Glucose tolerance tests
ITT: Insulin tolerance tests
LDL-C: Low-density lipoprotein cholesterol
AST: Aspartate transaminase
ALT: Alanine aminotransferase
T2DM: Type 2 diabetes mellitus
NAFLD: Nonalcoholic fatty liver disease
UC-MSC: Umbilical cord Wharton’s jelly-derived mesenchymal stem cells
PU-MSC: Dental pulp-derived mesenchymal stem cells
AD-MSC: Adipose tissue-derived MSC
